# Socioeconomic position and risk of unplanned hospitalization among nursing home residents: a nationwide cohort study

**DOI:** 10.1093/eurpub/ckaa207

**Published:** 2021-01-11

**Authors:** Katharina Allers, Amaia Calderón-Larrañaga, Stefan Fors, Lucas Morin

**Affiliations:** 1 Aging Research Center, Karolinska Institutet and Stockholm University, Stockholm, Sweden; 2 Department of Health Services Research, Carl von Ossietzky University of Oldenburg, Oldenburg, Germany; 3 Department of Medical Epidemiology and Biostatistics, Karolinska Institutet, Stockholm, Sweden; 4 Inserm CIC 1431, Clinical Investigation Unit, CHU Besançon, Besançon, France

## Abstract

**Background:**

Socioeconomic inequalities in health and healthcare use in old age have been on the rise during the past two decades. So far, it is unknown whether these inequalities have permeated the nursing home setting. This study aimed to assess whether the socioeconomic position of newly admitted nursing home residents had an influence on their risk of unplanned hospitalization.

**Methods:**

We identified older persons (≥75 years) who were newly admitted to a nursing home between March 2013 and December 2014 using a set of linked routinely collected administrative and healthcare data in Sweden. The number of unplanned hospitalizations for any cause and the cumulative length of stay were defined as primary outcomes. Unplanned hospitalizations for potentially avoidable causes (i.e. fall-related injuries, urinary tract infections, pneumonia and decubitus ulcers) were considered as our secondary outcome.

**Results:**

Among 40 545 newly admitted nursing home residents (mean age 86.8 years), the incidence rate of unplanned hospitalization ranged from 53.9 per 100 person-years among residents with tertiary education up to 55.1 among those with primary education. After adjusting for relevant confounders, we observed no meaningful difference in the risk of unplanned hospitalization according to the education level of nursing home residents (IRR for tertiary vs. primary education: 0.96, 95% CI 0.92–1.00) or to their level of income (IRR for highest vs. lowest quartile of income: 0.98, 0.95–1.02). There were also no differences in the cumulative length of hospital stays or in the risk of experiencing unplanned hospitalizations for potentially avoidable causes.

**Conclusions:**

In sum, in this large cohort of newly admitted nursing home residents, we found no evidence of socioeconomic inequalities in the risk of unplanned hospitalization.

## Introduction

Providing equal access to health care based on needs is a tenet of universal health care systems, such as in Sweden.^1^ Yet, socioeconomic inequalities in health and healthcare utilization have been on the rise over the past two decades, with particularly disquieting figures among older people.^2^^,^^3^ Previous reforms of the Swedish health care system, such as the freedom of choice regarding care providers,^4^ the reduction of the number of hospital beds,^5^ the marketization of long-term care^6^ and the increased threshold for entering institutional care facilities^7^ may have contributed to widening these socioeconomic inequalities.^8^

Unplanned hospitalizations are an interesting marker of the quality and continuity of care in nursing homes.^9^ These transfers often take a substantial toll on older people’s physical and cognitive functions. Among frail individuals with already depleted homeostatic reserves, they can trigger a downward spiral of adverse outcomes (e.g. decline in mobility, hospital-acquired infections, clinical complications, adverse drug reactions, injurious falls and even death).^10^ Despite conflicting results and varying degrees of quality of evidence, a large majority of published studies show that, in the community, older people with lower socioeconomic position are at greater risk of unplanned hospitalization.^11–16^ However, whether these inequalities have permeated the nursing home setting—where continuity of care is available to all residents regardless of their economic resources and social network—remains unknown.^12^^,^^17^

Inequalities in the accessibility of acute care and treatments seem unlikely in the context of the Swedish healthcare system,^18^ but other mechanisms may be at play. Differences in health literacy or in the attitudes and beliefs towards care among nursing home residents and their relatives could, for instance, serve as predisposing factors to explain variations in the propensity of individuals to request and accept non-elective procedures.^12^^,^^17^ Spatial-social segregation may also play a substantial role. The concentration of smaller-than-average nursing home facilities in rural and socially deprived geographical areas of the country could lead to higher rates of unplanned hospitalizations among residents with low socioeconomic position because of organizational issues (e.g. lack of trained geriatricians, absence of on-site nurses during nightshifts) rather than individual-level factors. Finally, the healthcare needs of nursing home residents with low and high levels of socioeconomic position may differ substantially at the time of nursing home admission, which would then be reflected in their respective patterns of healthcare use. In other words, socioeconomic disparities in unplanned hospitalizations could, if observed, be in large parts explained by differences in the underlying risk of developing clinical complications or experiencing unpredictable health events that warrant immediate attention by hospital specialists.

This study aimed to evaluate whether, in Sweden, the socioeconomic position of newly admitted nursing home residents had a causal influence on their subsequent risk of unplanned hospitalization. We hypothesized that nursing home residents with low and high socioeconomic position would present similar risks of unplanned hospitalization, and that any observed difference would be fully explained by socioeconomically patterned variations in health status at the time of nursing home admission.

## Methods

### Design, data source and study population

We used routinely collected data with full-population coverage in Sweden to assemble a longitudinal cohort study of newly admitted nursing home residents. Deterministic record-linkage was done by using unique pseudonymized identifiers (>99.9% accuracy).^19^ Individuals aged 75 years and older alive during the inclusion period, namely between March 2013 and December 2014, were identified in the Total Population Register.^20^ Data were then linked to the Social Services Register, which contains detailed information about the provision of municipal services to older persons, including home care help and nursing home admissions.

To exclude older adults who were already living in nursing homes before the inclusion period, we constructed a ‘washout’ period based on all available data points prior to March 2013. We then selected all individuals who had a first nursing home admission during the inclusion period and, after record-linkage with the Swedish Register of Education, excluded residents with missing data regarding educational attainment (*n*=858/48 799) and those with implausible inpatient records (*n*=5). Similarly, nursing home residents who died within the first 3 months after their admission were not included in the main analyses because older adults institutionalized at the end of life differ substantially from the general population of nursing home residents.

The date of cohort entry (‘time zero’) was defined as the date of nursing home admission. However, since the Social Services Register collects data about long-term care that reflects the situation of older people on the last day of every month, the month but not the exact day of nursing home admission was known to us. The date of cohort entry was, therefore, approximated by using the first day of the month immediately after nursing home admission (e.g. 1 April for a person admitted in March). Follow-up spanned from the date of cohort entry until either the occurrence of a censoring event (date of nursing home discharge, death and emigration) or the end of the available observation period (31 December 2015), whichever occurred first. Study design is represented graphically in Supplementary appendix eFigure S1.

### Socioeconomic position

Socioeconomic position was approximated with the highest educational attainment of individuals at the time of admission. This methodological choice was motivated by our hypothesis that health literacy rather than financial resources may potentially have an influence on the risk of unplanned hospitalization.^21^ The level of education was determined through data linkage with the Swedish Register of Education, which contains data from the 1970, 1975, 1980, 1985 and 1990 population and housing censuses, as well as annual updates from Statistics Sweden from 2000 to 2014 based on more than 30 different sources of information (e.g. compulsory schools, universities and agriculture schools). Data in the Education Register are adapted to the International Standard Classification of Education.^22^ The level of education was categorized as ‘primary’, ‘secondary’ and ‘tertiary’, in keeping with international standards (Supplementary appendix eTable S1). In *post-hoc* analyses, we operationalized socioeconomic position by using quartiles of disposable income (see Supplementary appendix for details).

### Outcomes

We defined the number of unplanned hospitalizations and the cumulative length of stay (in days) of unplanned hospitalizations during follow-up as primary outcomes, based on data from the National Patient Register.^23^ These include all emergency department visits and non-elective inpatient hospitalizations irrespective of the length of stay, that is including hospitalizations without overnight stay. Hospitalizations with overlapping dates of admission and discharge or separated by a single day were concatenated and counted as single events in order to avoid overestimating the number of admissions. Unplanned hospitalizations for fall-related injuries, urinary tract infections, pneumonia and bronchitis and decubitus ulcers—four conditions deemed potentially avoidable^9^—were defined as our secondary outcomes (Supplementary appendix eTable S2).

### Measurement of individual characteristics

Baseline characteristics of newly admitted nursing home residents were assembled based on available data in various national registers. Sex, age at time of nursing home admission, marital status and country of birth were retrieved from the Total Population Register. Physical frailty was approximated by using the Hospital Frailty Risk Score recently proposed by Gilbert et al.^24^ based on ICD-10 diagnosis codes reported during inpatient hospitalizations and specialized outpatient care visits in the 5 years before baseline (Supplementary appendix eTable S3). The burden of chronic multimorbidity was assessed by applying the methodology developed by Calderón-Larrañaga et al.^25^ Briefly, we captured a total of 56 long-lasting diseases and conditions defined as ‘chronic’ either because they leave substantial residual disability or impaired quality of life or because they result in a prolonged period of care, treatment and rehabilitation (Supplementary appendix eTable S4). Finally, we constructed three indicators of healthcare utilization: number of unplanned hospitalizations and number of days of inpatient stay during the year before nursing home admission, and number of prescribed drugs during the week before.

### Statistical analysis

Baseline characteristics across socioeconomic groups are reported with standard descriptive methods. The incidence rate of unplanned hospitalizations during follow-up is reported per 100 person-years and was calculated by dividing the total number of such events by the number of person-years at-risk. To avoid immortal time bias, the cumulative length of inpatient hospitalizations during follow-up was subtracted from the total time under observation. The average number of days of hospitalization per person-years was calculated by considering the total time under observation as time at-risk. We used zero-inflated Poisson regression models to compare the risk of unplanned hospitalization between residents with primary, secondary and tertiary education. Zero-inflated Poisson regression was chosen as our primary analytical approach because a substantial share of newly admitted nursing home residents had no unplanned hospitalization during follow-up (i.e. an excess of zero counts). We calculated the incidence risk ratios (IRRs) while adjusting on available relevant confounders, which were selected based on causal diagrams informed by previous research and subject-matter knowledge.^26^ Analyses were thereafter adjusted on sex, age, marital status, physical frailty, number of chronic diseases, number of prescription drugs and cumulative length of inpatient stay during the year before nursing home admission. The number of chronic diseases and the length of inpatient stay before nursing home admission were also included in the ‘zero’ model. The appropriateness of using zero-inflated models was evaluated graphically, first by plotting the quantiles of the observed values against the quantiles of normal distribution (‘normality QQ plot’) and second by plotting the individual predicted probabilities under the zero-inflated model and the Poisson model against each other (i.e. departure from the *x=y* line indicates that zero-inflation is preferable).^27^ We also used the Akaike’s information criterion to verify that the zero-inflated Poisson model provided a better fit than its non-zero alternative. Analyses were repeated with zero-inflated negative binomial regressions to assess the robustness of our estimates to various modelling assumptions. IRRs are reported with their 95% confidence intervals (CI). We conducted a comprehensive set of prespecified sensitivity analyses and non-prespecified *post-hoc* analyses to confirm the robustness of our findings to different methodological assumptions (see Supplementary appendix for details).

Statistical analyses were performed using SAS version 9.4 (SAS Institute Inc., Cary, NC). Statistical code was programmed by a single author (K.A.) but was then reviewed independently by a second author (L.M.) for quality control. In addition, all analyses were replicated independently on Stata version 16.0 (StataCorp, College Station, TX), to identify potential sources of bias in the operationalization of the study protocol and thus enhance reproducibility.

### Ethical approval

The Regional Ethical Review Board in Stockholm approved the study. The need for individual consent was waived since the study relies exclusively on routinely collected administrative and healthcare data.

## Results

### Characteristics of the study population

Of all 48 799 newly admitted nursing home residents during the study period, 40 545 (83%) persons met our inclusion criteria (Supplementary appendix eFigure S2). Mean age at baseline was 86.8 (SD 5.5) years, and 66.1% of these residents were female. Overall, 20 614 (50.8%) residents had attained primary education, 15 339 (37.8%) secondary education and 4592 (11.3%) tertiary education. Residents with higher education were more often male, more often married, had a higher physical frailty score, and were prescribed a lower number of drugs than those with primary education (table 1). There was no qualitatively important difference in the number of chronic diseases and the number or length of unplanned hospitalizations during the year before nursing home admission. Moreover, the mortality rate during follow-up was similar across levels of education (Supplementary appendix eTable S5).

**Table 1:** Baseline characteristics of newly admitted nursing home residents

	Total	Primary education	Secondary education	Tertiary education
Number of nursing home residents	40 545	20 614 (50.8%)	15 339 (37.8%)	4592 (11.3%)
Sex, *N* (%)				
Men	13 738 (33.9%)	6652 (32.3%)	5116 (33.4%)	1970 (42.9%)
Women	26 807 (66.1%)	13 962 (67.7%)	10 223 (66.6%)	2622 (57.1%)
Age at baseline, years				
Mean (SD)	86.8 (5.5)	87.3 (5.4)	86.3 (5.6)	86.0 (5.6)
*N* (%)				
75–84 years	15 202 (37.5%)	6931 (33.6%)	6259 (40.8%)	2012 (43.8%)
85–94 years	22 806 (56.2%)	12 258 (59.5%)	8213 (53.5%)	2335 (50.9%)
≥95 years	2537 (6.3%)	1425 (6.9%)	867 (5.7%)	245 (5.3%)
Marital status, *N* (%)				
Married	10 125 (25.0%)	4617 (22.4%)	3979 (25.9%)	1529 (33.3%)
Single or divorced	8003 (19.7%)	3688 (17.9%)	3309 (21.6%)	1006 (21.9%)
Widowed	22 417 (55.3%)	12 309 (59.7%)	8051 (52.5%)	2057 (44.8%)
Country of birth, *N* (%)				
Sweden	36 961 (91.2%)	19 031 (92.3%)	13 752 (89.7%)	4178 (91.0%)
Foreign-born	3584 (8.8%)	1583 (7.7%)	1587 (10.3%)	414 (9.0%)
Number of chronic diseases				
Mean (SD)	6.9 (3.6)	6.8 (3.5)	7.0 (3.6)	7.0 (3.6)
Number of prescribed drugs during the week before				
Mean (SD)	7.3 (3.9)	7.4 (3.9)	7.3 (4.0)	6.8 (3.9)
*N* (%)				
0–4 drugs	10 218 (25.2%)	4987 (24.2%)	3918 (25.5%)	1313 (28.6%)
5–9 drugs	19 472 (48.0%)	9946 (48.2%)	7311 (47.7%)	2215 (48.2%)
≥10 drugs	10 855 (26.8%)	5681 (27.6%)	4110 (26.8%)	1064 (23.2%)
Hospital frailty risk score				
Median (first and third quartiles)	7.6 (3.2–13.1)	6.8 (2.9–12.1)	8.2 (3.7–13.8)	9.0 (4.1–14.7)
*N* (%)				
Low (<5)	14 213 (35.1%)	7898 (38.3%)	4937 (32.2%)	1378 (30.0%)
Moderate (5–14)	18 779 (46.3%)	9441 (45.8%)	7234 (47.2%)	2104 (45.8%)
High (≥15)	7553 (18.6%)	3275 (15.9%)	3168 (20.6%)	1110 (24.2%)
Unplanned hospitalizations during the year before				
Mean (SD)	1.3 (1.4)	1.3 (1.4)	1.3 (1.4)	1.3 (1.4)
*N* (%)				
0	12 790 (31.5%)	6455 (31.3%)	4828 (31.5%)	1507 (32.8%)
1	14 215 (35.1%)	7295 (35.4%)	5361 (35.0%)	1559 (34.0%)
2	7384 (18.2%)	3734 (18.1%)	2797 (18.2%)	853 (18.6%)
≥3	6156 (15.2%)	3130 (15.2%)	2353 (15.3%)	673 (14.7%)
Cumulative length of inpatient stay during the year before				
Mean (SD)	16.4 (20.7)	15.9 (19.6)	17.1 (21.7)	16.7 (21.7)
*N* (%)				
0 days	12 019 (29.6%)	6111 (29.6%)	4514 (29.4%)	1394 (30.4%)
1–7 days	4863 (12.0%)	2496 (12.1%)	1804 (11.8%)	563 (12.3%)
8–14 days	7193 (17.7%)	3792 (18.4%)	2649 (17.3%)	752 (16.4%)
15–29 days	8863 (21.9%)	4562 (22.1%)	3318 (21.6%)	983 (21.4%)
≥30 days	7607 (18.8%)	3653 (17.7%)	3054 (19.9%)	900 (19.6%)
Vital status at the end of follow-up				
Alive	24 208 (59.7%)	12 077 (58.6%)	9336 (60.9%)	2795 (60.9%)
Dead	16 331 (40.3%)	8532 (41.4%)	6002 (39.1%)	1797 (39.1%)
Emigrated	6 (0.0%)	5 (0.0%)	1 (0.0%)	0 (0.0%)

### Incidence of unplanned hospitalizations

The overall incidence rate of unplanned hospitalization was 55.1 per 100 person-years, ranging from 55.1 (95% CI 54.3–55.9) among residents with primary education down to 53.9 (95% CI 52.2–55.7) per 100 person-years among residents with tertiary education (table 2). There was no difference in the adjusted risk of unplanned hospitalization between residents with a primary or secondary education (IRR 0.99, 95% CI 0.96–1.02) and only a small and statistically non-significant decrease among residents with a tertiary education (IRR 0.96, 95% CI 0.92–1.00). These results remained similar when using Poisson or zero-inflated negative binomial regression models (Supplementary appendix eTable S6). Operationalizing socioeconomic position based on income instead of education did not change the results in a scientifically important manner (table 3). There was, for instance, no difference in the risk of unplanned hospitalization between residents in the lowest and highest income quartiles (IRR 0.98, 95% CI 0.95–1.02).

**Table 2:** Level of education and risk of unplanned hospitalization among newly admitted nursing home residents

	Primary education	Secondary education	Tertiary education
Number of nursing home residents	20 614	15 339	4592
Unplanned hospitalizations			
Person-years of follow-up	30 169	22 429	6660
Number of events	16 621	12 425	3592
Rate per 100 person-years (95% CI)	55.1 (54.3–55.9)	55.4 (54.4–56.4)	53.9 (52.2–55.7)
Unadjusted incidence rate ratio (95% CI)	1	1.01 (0.98–1.04)	1.00 (0.96–1.04)
Adjusted incidence rate ratio (95% CI)^a^	1	0.99 (0.96–1.02)	0.96 (0.92–1.00)
*E*-value^b^		1.11	1.25
Cumulative length of stay of unplanned hospitalizations			
Person-years of follow-up	30 465	22 658	6724
Total number of days hospitalized	101 846	78 406	21 941
Fraction of time spend in hospital (%)	0.9%	0.9%	0.9%
Number of days hospitalized per person-year (95% CI)	3.3 (3.3–3.4)	3.5 (3.4–3.5)	3.3 (3.2–3.3)
Unadjusted incidence rate ratio (95% CI)	1	1.05 (1.04–1.06)	1.04 (1.02–1.05)
Adjusted incidence rate ratio (95% CI)^a^	1	1.03 (1.02–1.04)	1.01 (0.99–1.02)
*E*-value^b^		1.21	1.11

a Zero-inflated Poisson regression model adjusted for age, sex, marital status, frailty, number of chronic diseases, number of drugs and number of days of inpatient stay before nursing home admission. The ‘zero’ model was adjusted for the number of chronic diseases and the number of days of inpatient stay before nursing home admission.

b *E*-values are reported for point estimates. Interpretation: we found that the observed adjusted IRR for unplanned hospitalization of 0.96 (tertiary vs. primary education) could be fully explained away by an unmeasured confounder that was associated with both education and risk of unplanned hospitalization by an IRR of 1.25 either above and beyond the measured confounders, but a weaker confounder could not do so.

95% CI, 95% confidence interval.

**Table 3:** Income and risk of unplanned hospitalization among newly admitted nursing home residents

	Lowest income quartile	Second income quartile	Third income quartile	Highest income quartile
Number of nursing home residents	9324	10 375	11 003	9 821
Person-years of follow-up	13 936	15 291	15 886	14 115
Number of unplanned hospitalizations	7465	8403	9145	7 603
Rate per 100 person-years (95% CI)	53.6 (52.4–54.8)	55.0 (53.8–56.1)	57.6 (56.4–58.8)	53.9 (52.7–55.1)
Unadjusted incidence rate ratio (95% CI)	1	1.02 (0.98–1.06)	1.08 (1.04–1.11)	1.01 (0.97–1.05)
Adjusted incidence rate ratio (95% CI)^a^	1	1.00 (0.96–1.03)	1.03 (0.99–1.06)	0.98 (0.95–1.02)

A total of 22 individuals had no data regarding their annual income and were thus excluded from the analysis. Income quartiles were calculated for each sex–age stratum separately. 95% CI, 95% confidence interval.

a Zero-inflated Poisson regression model adjusted for age, sex, marital status, frailty, number of chronic diseases, number of drugs and number of days of inpatient stay before nursing home admission. The ‘zero’ model was adjusted for the number of chronic diseases and the number of days of inpatient stay before nursing home admission.

### Cumulative length of stay of unplanned hospitalizations

The number of days spent in hospital for unplanned hospitalizations accounted for 0.9% of the total contributing time of nursing home residents, irrespective of their level of education (table 2). We observed no difference in the risk of spending more days in hospital between the highest and lowest level of education (IRR 1.01, 95% CI 0.99–1.02). Non-zero-inflated Poisson regression provided similar albeit slightly lower estimates (Supplementary appendix eTable S7).

### Unplanned hospitalizations for potentially avoidable causes

As shown in table 4, the risk of cause-specific unplanned hospitalizations did not vary according to the level of education of newly admitted nursing home residents. Hence, after adjusting for known confounders, residents who had a tertiary education had a similar risk of experiencing unplanned hospitalization for urinary tract infections, pneumonia and bronchitis, decubitus ulcers or fall-related injuries.

**Table 4:** Level of education and risk of cause-specific unplanned hospitalization among newly admitted nursing home residents

	Primary education	Secondary education	Tertiary education
Number of nursing home residents	20 614	15 339	4592
Urinary tract infections			
Person-years of follow-up	30 169	22 429	6660
Number of unplanned hospitalization	2113	1645	483
Rate per 100 person-years (95% CI)	7.0 (6.7–7.3)	7.3 (7.0–7.7)	7.3 (6.6–7.9)
Adjusted incidence rate ratio (95% CI)^a^	1	1.03 (0.95–1.10)	0.99 (0.88–1.11)
*E*-value^b^		1.21	1.11
Pneumonia and bronchitis			
Person-years of follow-up	30 169	22 429	6660
Number of unplanned hospitalization	2473	1806	620
Rate per 100 person-years (95% CI)	8.2 (7.9–8.5)	8.1 (7.7–8.4)	9.3 (8.6–10.1)
Adjusted incidence rate ratio (95% CI)^c^	1	0.97 (0.90–1.04)	1.07 (0.96–1.19)
*E*-value^b^		1.21	1.34
Decubitus ulcers			
Person-years of follow-up	30 169	22 429	6660
Number of unplanned hospitalization	154	126	38
Rate per 100 person-years (95% CI)	0.5 (0.4–0.6)	0.6 (0.5–0.7)	0.6 (0.4–0.8)
Adjusted incidence rate ratio (95% CI)^d^	1	0.99 (0.76–1.29)	0.90 (0.59–1.36)
*E*-value^b^		1.11	1.46
Falls and fall-related injuries			
Person-years of follow-up	30 169	22 429	6660
Number of unplanned hospitalization	3199	2392	654
Rate per 100 person-years (95% CI)	10.6 (10.2–11.0)	10.7 (10.2–11.1)	9.8 (9.1–10.6)
Adjusted incidence rate ratio (95% CI)^e^	1	1.03 (0.97–1.09)	0.95 (0.87–1.04)
*E*-value^b^		1.21	1.29

a Zero-inflated Poisson regression model adjusted for age, sex, marital status, frailty, number of chronic diseases (excluding diabetes), number of drugs and diabetes. The ‘zero’ model was adjusted for the number of chronic diseases.

b *E*-values are reported for point estimates. Interpretation: we found that the observed adjusted IRR for unplanned hospitalization for urinary tract infection of 0.99 (tertiary vs. primary education) could be fully explained away by an unmeasured confounder that was associated with both education and risk of unplanned hospitalization by an IRR of 1.11 either above and beyond the measured confounders, but a weaker confounder could not do so.

c Zero-inflated Poisson regression model adjusted for age, sex, marital status, number of chronic diseases, number of drugs, prescription of corticosteroids (ATC code H02), immunosuppressants (ATC code L04) and proton pump inhibitors (ATC code A02BC) and dementia. The ‘zero’ model was adjusted for the number of chronic diseases.

d Zero-inflated Poisson regression model adjusted for age, sex, marital status, frailty, number of chronic diseases, history of decubitus ulcer, number of drugs and number of days of inpatient stay before nursing home admission. The ‘zero’ model was adjusted for the number of chronic diseases and the number of days of inpatient stay before nursing home admission.

e Zero-inflated Poisson regression model adjusted for age, sex, marital status, frailty, number of chronic diseases, number of drugs, number of days of inpatient stay before nursing home admission and history of falls and fall-related injuries in the year prior to nursing home admission. The ‘zero’ model was adjusted for the number of chronic diseases and the number of days of inpatient stay before nursing home admission.

95% CI, 95% confidence interval.

### Sensitivity analyses

Results of the sensitivity analyses are provided in Supplementary appendix eTables S8–S14. Our findings did not change when restricting the analysis to people admitted in or after June 2013 (6-months wash-out period). Also, limiting the analysis to nursing home residents with complete and uninterrupted follow-up data in the Social Services Register did not modify the association between education and risk of unplanned hospitalization. Including people who died within the first three months after nursing home admission or on the contrary restricting the analysis to residents who survived at least 6 months did also not meaningfully change the findings, neither did moving the date of nursing home admission to 15 days before. As shown in Supplementary eTable S8, residents with higher-than-primary education had a higher probability of planned (i.e. elective) hospitalization. *E*-value analysis showed that the observed reduced risk of unplanned hospitalization of 0.96 for residents with tertiary education compared to those with primary education could be fully explained away by an unmeasured confounder that was associated with both education and risk of unplanned hospitalization by a magnitude of 1.25 both above and beyond the measured confounders, but a weaker confounder could not do so. In non-prespecified *post-hoc* analyses, we found a modest and statistically non-significant multiplicative interaction between higher education and high-income quartile (Supplementary eTable S15). Subgroup analyses by sex revealed that while no educational difference in the risk of unplanned hospitalizations could be detected among men, a modest decrease was observed among women with tertiary education (IRR 0.93, 95% CI 0.88–0.98; Supplementary eTable S16).

## Discussion

In this nationwide study of newly admitted nursing home residents in Sweden, we found no scientifically important or clinically relevant difference in the risk of unplanned hospitalization between residents with lower or higher socioeconomic position (whether approximated by education or income). There was also no difference in the cumulative length of hospital stay or in the risk of experiencing unplanned hospitalizations for causes deemed potentially avoidable. Our findings were robust to various assumptions and analytical strategies.

The observed incidence rate of unplanned hospitalization of 55.1 per 100 person-years is within the range of international findings.^28–30^ Our finding of a similar risk of unplanned hospital admission among residents with different levels of education is in line with another Swedish study on transitions between care settings at the end of life. Hence, Kelfve et al.^14^ found that a lower level of education was associated with a higher likelihood of dying in the hospital among older community-dwellers, whereas no educational difference was observed among nursing home residents. In contrast, a study from Norway reported that a higher level of education was correlated with a higher risk of hospital admission among cognitively intact nursing home residents.^13^

We hypothesized that differences in the risk of unplanned hospitalization could arise due to socioeconomically patterned variations in health status at the time of nursing home admission. Indeed, nursing home residents with a higher level of education were found to have a higher risk of physical frailty at baseline, which suggests that older people with different socioeconomic position are admitted to nursing homes for different reasons and, thus, that disparities in subsequent health care utilization may occur due to different care needs rather than because of inequity. After adjusting for a wide range of health-related covariates, such as physical frailty, comorbidities, prescribed drugs and previous hospital admissions, the risk of unplanned hospitalizations was strikingly similar across educational groups. Of note, while no educational difference in the risk of unplanned hospital admission was found among men, we detected a modest decrease among women with tertiary education. Whether this indicates a true gender difference or stems from residual confounding specific to women should be further investigated in future studies.

It is likely that the residents’ preferences and wishes regarding medical care would vary according to their socioeconomic position. For instance, the existing literature shows a greater uptake of advance care planning among people with higher education.^12^ Direct information about these preferences and wishes were not available in this study. However, we found that planned hospitalizations were substantially more frequent among residents with higher-than-primary education. This phenomenon, which has previously been described in the community setting,^11^ is thought to be the result of the greater propensity for older people with higher socioeconomic position to adopt a more ‘proactive’ approach to tertiary care and elective procedures. Our study therefore suggests that although differences in preferences and attitudes towards care exist among nursing home residents with difference socioeconomic backgrounds, these preferences do not apply to unplanned hospitalizations.

One possible explanation for the absence of influence of socioeconomic position on the risk of unplanned hospitalization is that while people living at home decide on their own or with their relatives when and in which way to contact the health care system, nursing home professionals act as gatekeepers for outpatient and hospital care.^31^ In addition, the living conditions of nursing home residents (e.g. housing, diet and drug administration) are more standardized than in the community setting. Nursing homes may thus have an ‘equalizing effect’ and smooth out the socioeconomic gradient seen in the community. However, although residents were equally likely to be hospitalized irrespective of their level of education, the generally high rates of unplanned hospitalization raise serious concern. These transitions are often avoidable through timely and appropriate care in the nursing homes.^32^^,^^33^ As suggested in previous studies, interventions to prevent falls, adequate staff-resident-ratio, improvements in professional capacity and widespread use of advance care planning can help reduce the number of avoidable hospitalizations.^34^^,^^35^

This study is based on high-quality routinely collected data with nationwide coverage in Sweden, which enabled us to assemble a large inception cohort of newly admitted nursing home residents with nearly complete data regarding education (<2% missing values). However, the following limitations have to be considered. First, due to the observational nature of this study, we cannot rule out the existence of unmeasured confounders that may distort the observed association between socioeconomic position and unplanned hospitalization. Information about cognition, physical function and disability were, for instance unavailable. *E*-value analysis showed that unmeasured confounders of low to moderate magnitude (1.11–1.34) would fully explain away the very modest and statistically non-significant effect sizes observed in this study. In our opinion such unmeasured confounders are very likely to exist, which reinforces our conclusion that there are no socioeconomic disparities in the risk of unplanned hospitalization in nursing homes. Second, studies have shown that people with a lower level of education have a lower life expectancy this might have led to a ‘healthy survivor’ selection bias (i.e. informative left truncation). Third, the exact date of nursing home admission was unknown to us, which could have led to a certain degree of inaccuracy. However, sensitivity analyses showed a remarkable stability of our findings regardless of the choice of cohort entry date. Fourth, available data did not include information about nursing home characteristics. Facility-level factors have been found to play an important role in the occurrence of unplanned hospitalizations.^36^ Hence, the resident–staff ratio, the available medical equipment^37^ or the type of care provider^38^ may act as ‘effect modifiers’ and magnify or on the contrary buffer socioeconomic inequalities between nursing home residents. Finally, following the 2010 reform giving older people the freedom to choose their (long-term) care providers, residents with better health literacy are more likely to choose nursing homes facilities that meet their needs and expectations.^39^ This may generate social and economic health inequalities that our study could not shed light on.

A primary aim of many health care systems is to provide equal access to care. Nursing home residents are an especially vulnerable group with high care needs. Taken together, our results suggest that the socioeconomic inequalities in unplanned hospital admissions that have been reported among community-dwellers may not extend to newly admitted nursing home residents. Qualitative studies would be well suited to unravel the putative mechanisms behind this apparent absence of socioeconomic disparities. Epidemiological studies are also needed to investigate whether inequalities in nursing homes exist in other outcomes, such as comfort and palliative care. Moreover, despite the absence of a socioeconomic gradient, the rate of unplanned hospitalizations among nursing home residents is worryingly high. This points to the need for better coordination between nursing home staff and acute and palliative care providers.

## Supplementary data

Supplementary data are available at *EURPUB* online.

## Funding

This study did not receive any specific grant from funding agencies in the public, commercial, or not-for-profit sectors.

## Disclaimer

All authors gave approval for the final version of the manuscript and agree to be accountable for all aspects of the work.

## Ethical approval

The study was approved by the Regional Ethical Review Board in Stockholm.

## Data availability

Clinical data cannot be made publicly available because of privacy issues. However, additional results and aggregated findings are available on reasonable request.

*Conflicts of interest*: None declared.

**Key points**

- So far, it was unknown whether socioeconomic inequalities affect care provided in the nursing home setting.
- In this study, unplanned hospitalizations were used as a marker for the quality of care.
- Overall, the rate of unplanned hospitalizations among nursing home residents is elevated.
- We found no evidence of socioeconomic inequalities among nursing home residents.
- Nursing homes may serve as a ‘equalizer’ to smooth down socioeconomic disparities.

## Supplementary Material

ckaa207_Supplementary_Data
