# Supplementary material for: Socioeconomic position and risk of unplanned hospitalization among nursing home residents: a nationwide cohort study
Source: Eur J Public Health. 2021 Jan 11;31(3):467–73. doi: 10.1093/eurpub/ckaa207 (PMC8495905; doi:10.1093/eurpub/ckaa207)
Supplement: ckaa207_Supplementary_Data [file ckaa207_supplementary_data.docx]

# Appendix

eFigure 1. Study design

eFigure 2. Flowchart of the study population

eTable 1. Categorization of educational attainment

eTable 2. ICD-10 codes to assess cause-specific unplanned hospitalisations

eTable 3. ICD-10 codes to assess the hospital frailty risk

eTable 4. ICD-10 and ATC codes to assess number of chronic diseases

eTable 5. Mortality rates by level of education

eTable 6. Sensitivity analysis: level of education and risk of unplanned hospitalisation among newly admitted nursing home residents

eTable 7. Sensitivity analysis: level of education and cumulative length of stay of unplanned hospitalisations

eTable 8. Sensitivity analysis: level of education and risk of planned hospitalisation among newly admitted nursing home residents

eTable 9. Sensitivity analysis: income quartile and risk of unplanned hospitalisation among newly admitted nursing home residents (Poisson regression)

eTable 10. Sensitivity analysis: level of education and risk of unplanned hospitalisations among nursing home residents admitted in or after June 2013 (6-month washout)

eTable 11. Sensitivity analysis: level of education and risk of unplanned hospitalisations among nursing home residents with complete and uninterrupted follow-up data in the Social Services Register

eTable 12. Sensitivity analysis: level of education and risk of unplanned hospitalisation during follow-up among all newly admitted residents (including those who died within 3 months)

eTable 13. Sensitivity analysis: level of education and risk of unplanned hospitalisation during follow-up among nursing home residents who survived at least 6 months (Poisson regression)

eTable 14. Sensitivity analysis: level of education and risk of unplanned hospitalisation during follow-up, with mid-month approximation of the date of admission

eTable 15. Multiplicative interaction effects between education and income, sex, age and frailty

eTable 16. Subgroup analysis: level of education and risk of unplanned hospitalisation among newly admitted nursing home residents, stratified by sex

**eFigure 1.Study design**


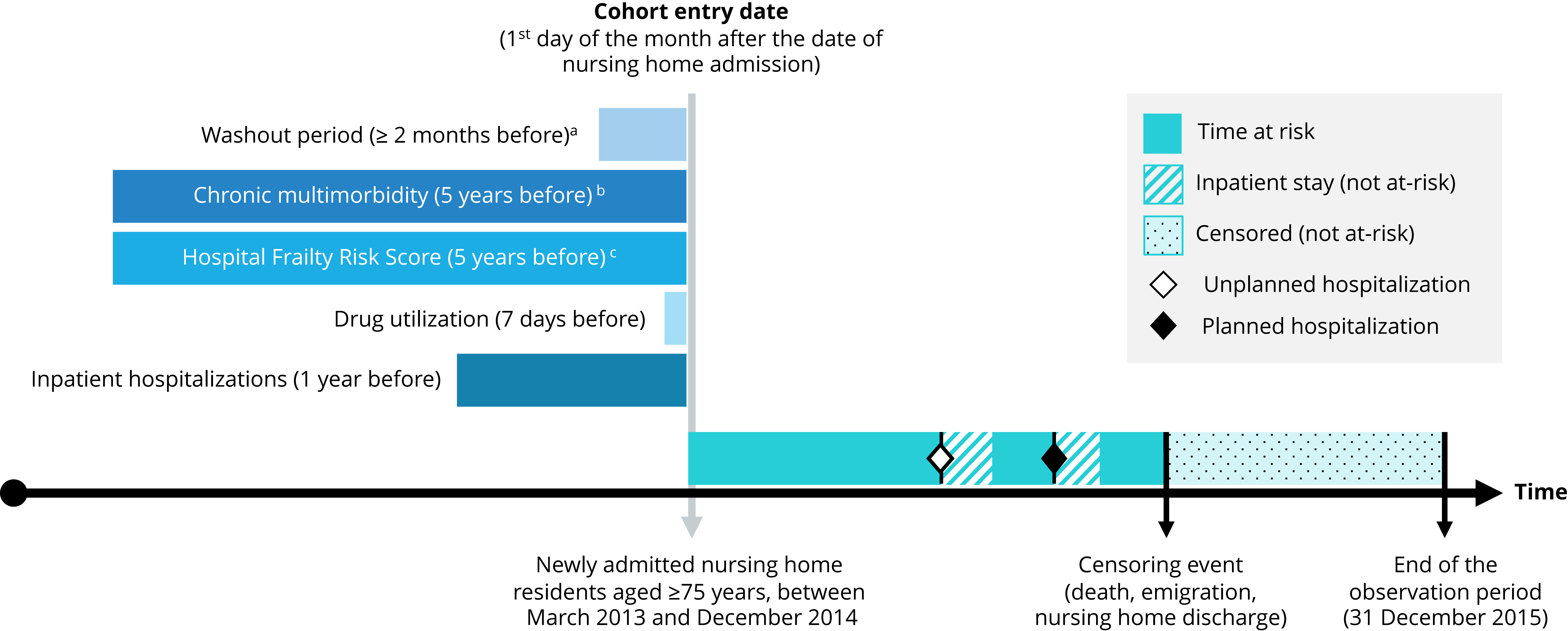


This graphical representation of the study design follows the recently proposed visualization framework from Schneeweiss S, et al. (*Ann Intern Med*. 2019;170[6]:398). In this hypothetical example, the person is followed-up from the day of cohort entry until the occurrence of a censoring event. During follow-up, this person experienced one unplanned hospitalisation (first co-primary outcome) and one planned hospitalisation (sensitivity analysis outcome). To avoid immortal time bias, days spent in hospitals are removed from the contributing time: by definition, study subjects cannot experience an unplanned hospitalisation during an inpatient stay. The number of days of inpatient stay following unplanned hospitalisations (second co-primary outcome) is represented by the first hashed rectangle. Here, time at-risk was defined as the entire time under observation.

*^a^ The washout period was constructed based on all available data points prior to March 2013. Data from the Swedish Social Services Register goes back to 2007, but during the period 2007–2012 information was only registered at irregular intervals (October 2007, June 2008, December 2008, June 2009, December 2009, November 2010, April 2011, October 2011, April 2012, and October 2012). Thus, combining these assessments with high-quality data from January and February 2013 enabled us to detect older adults who had already been admitted to nursing homes before the inclusion period.*

*^b^ Details about the assessment of chronic multimorbidity are presented in eTable 4*

*^c^ Details about the calculation of the Hospital Frailty Risk Score are presented in eTable 3*

**eFigure 2. Flowchart of the study population**

**eTable 1. Categorization of educational attainment**

| **Level of education** | **Categories** | **ISCED97** | **SUN2000** |
| --- | --- | --- | --- |
| Primary education | Primary education or first stage of basic education | 1 | 100 |
| Secondary education | Lower secondary education | 2A | 200-206 |
|  | Upper secondary education ≤ 2 years | 3C | 310-327 |
|  | Upper secondary education ≥3 years | 3A | 330–337 |
|  | Post-secondary, non-tertiary education <2 years | 4C, 4A | 410–417 |
| Tertiary education | Tertiary education for practical/ technical occupations | 5B | 520–527 |
|  | Tertiary or academic education giving access to professions with high skills requirements | 5A | 530–557 |
|  | Post-graduate education (licentiate/doctorate) | 6 | 600–640 |

**eTable 2. ICD-10 codes to assess cause-specific unplanned hospitalisations**

| **Condition** | **ICD-10 codes** |
| --- | --- |
| Falls and fall-related injuries  (according to Morin et al.) | W00–W19, S01, S02, S06, S07, S11, S12, S17, S21, S22, S28, S31, S32, S36, S38, S41, S42, S47, S51, S52, S57, S61, S62, S67, S71, S72, S77, S81, S82, S87, S91, S92, S97, T01, T02, T04, T08, T10, T11 |
| Urinary tract infection  (according to Muench et al.) | N10, N11, N110, N111, N118, N119, N15, N151, N1510, N1511, N158, N159, N16, N160, N161, N162, N163, N164, N165, N168, N291, N30, N300, N301, N302, N303, N304, N308, N309,  N330, N34, N340, N341, N342, N343, N351, N37, N370, N378,  N390 |
| Pneumonia and bronchitis  (according to Muench et al.) | J12, J120, J121, J122, J123, J128, J129, J13, J18, J180, J181, J182, J188, J189, J15, J150, J151, J152, J153, J154, J155, J156, J157, J158, J159, J14, A481, J16, J160, J168  J690 |
| Decubitus ulcer and pressure area (according to Sundmacher et al.) | L89 |

L. Morin, A. Calderon Larrañaga, A.-K. Welmer, D. Rizzuto, J. Wastesson, K. Johnell, Polypharmacy and injurious falls in older adults: a nationwide nested case-control study, *Clin. Epidemiol*. Volume 11 (2019) 483–493. doi:10.2147/CLEP.S201614.

U. Muench, M. Simon, R.-A. Guerbaai, C. De Pietro, A. Zeller, R.W. Kressig, F. Zúñiga, INTERCARE Research Group, Preventable hospitalisations from ambulatory care sensitive conditions in nursing homes: evidence from Switzerland, *Int. J. Public Health*. (2019). doi:10.1007/s00038-019-01294-1.

L. Sundmacher, D. Fischbach, W. Schuettig, C. Naumann, U. Augustin, C. Faisst, Which hospitalisations are ambulatory care-sensitive, to what degree, and how could the rates be reduced? Results of a group consensus study in Germany, *Health Policy*. 119 (2015) 1415–1423. doi:10.1016/j.healthpol.2015.08.007.

**eTable 3. ICD-10 codes to assess the hospital frailty risk**

The ﻿Hospital Frailty Risk Score was initially developed and validated for older adults in acute care settings. For the present study, it was computed based on ICD-10 codes reported in all inpatient and specialized outpatient care discharge reports during the 5 years before nursing home admission. For more information, please refer to: Gilbert T, Neuburger J, Kraindler J, et al. Development and validation of a Hospital Frailty Risk Score focusing on older people in acute care settings using electronic hospital records: an observational study. *Lancet*. 2018;391(10132):1775-1782.

| **#** | **ICD-10 code** | **Description** | **Weight** |
| --- | --- | --- | --- |
| 1 | F00 | Dementia in Alzheimer's disease | 7.1 |
| 2 | G81 | Hemiplegia | 4.4 |
| 3 | G30 | Alzheimer's disease | 4 |
| 4 | I69 | Sequelae of cerebrovascular disease (secondary codes) | 3.7 |
| 5 | R29 | Other symptoms and signs of the nervous and musculoskeletal systems | 3.6 |
| 6 | N39 | Other disorders of urinary system (e.g. urinary tract infection) | 3.2 |
| 7 | F05 | Delirium, not induced by alcohol and other psychoactive substances | 3.2 |
| 8 | W19 | Unspecified fall | 3.2 |
| 9 | S00 | Superficial injury of head | 3.2 |
| 10 | R31 | Unspecified haematuria | 3 |
| 11 | B96 | Other bacterial agents as the cause of diseases | 2.9 |
| 12 | R41 | Other symptoms and signs involving cognitive functions and awareness | 2.7 |
| 13 | R26 | Abnormalities of gait and mobility | 2.6 |
| 14 | I67 | Other cerebrovascular diseases | 2.6 |
| 15 | R56 | Convulsions, not elsewhere classified | 2.6 |
| 16 | R40 | Somnolence, stupor and coma | 2.5 |
| 17 | T83 | Complications of genitourinary prosthetic devices, implants and grafts | 2.4 |
| 18 | S06 | Intracranial injury | 2.4 |
| 19 | S42 | Fracture of shoulder and upper arm | 2.3 |
| 20 | E87 | Other disorders of fluid, electrolyte and acid-base balance | 2.3 |
| 21 | M25 | Other joint disorders, not elsewhere classified | 2.3 |
| 22 | E86 | Volume depletion | 2.3 |
| 23 | R54 | Senility | 2.2 |
| 24 | Z50 | Care involving use of rehabilitation procedures | 2.1 |
| 25 | F03 | Unspecified dementia | 2.1 |
| 26 | W18 | Other fall on same level | 2.1 |
| 27 | Z75 | Problems related to medical facilities and other health care | 2 |
| 28 | F01 | Vascular dementia | 2 |
| 29 | S80 | Superficial injury of lower leg | 2 |
| 30 | L03 | Cellulitis | 2 |
| 31 | H54 | Blindness and low vision | 1.9 |
| 32 | E53 | Deficiency of other B group vitamins | 1.9 |
| 33 | Z60 | Problems related to social environment | 1.8 |
| 34 | G20 | Parkinson's disease | 1.8 |
| 35 | R55 | Syncope and collapse | 1.8 |
| 36 | S22 | Fracture of rib(s), sternum and thoracic spine | 1.8 |
| 37 | K59 | Other functional intestinal disorders | 1.8 |
| 38 | N17 | Acute renal failure | 1.8 |
| 39 | L89 | Decubitus ulcer | 1.7 |

**eTable 3. Continued**

| **#** | **ICD-10 code** | **Description** | **Weight** |
| --- | --- | --- | --- |
| 40 | Z22 | Carrier of infectious disease | 1.7 |
| 41 | B95 | Streptococcus and staphylococcus | 1.7 |
| 42 | L97 | Ulcer of lower limb, not elsewhere classified | 1.6 |
| 43 | R44 | Other symptoms and signs involving general sensations and perceptions | 1.6 |
| 44 | K26 | Duodenal ulcer | 1.6 |
| 45 | I95 | Hypotension | 1.6 |
| 46 | N19 | Unspecified renal failure | 1.6 |
| 47 | A41 | Other septicaemia | 1.6 |
| 48 | Z87 | Personal history of other diseases and conditions | 1.5 |
| 49 | J96 | Respiratory failure, not elsewhere classified | 1.5 |
| 50 | X59 | Exposure to unspecified factor | 1.5 |
| 51 | M19 | Other arthrosis | 1.5 |
| 52 | G40 | Epilepsy | 1.5 |
| 53 | M81 | Osteoporosis without pathological fracture | 1.4 |
| 54 | S72 | Fracture of femur | 1.4 |
| 55 | S32 | Fracture of lumbar spine and pelvis | 1.4 |
| 56 | E16 | Other disorders of pancreatic internal secretion | 1.4 |
| 57 | R94 | Abnormal results of function studies | 1.4 |
| 58 | N18 | Chronic renal failure | 1.4 |
| 59 | R33 | Retention of urine | 1.3 |
| 60 | R69 | Unknown and unspecified causes of morbidity | 1.3 |
| 61 | N28 | Other disorders of kidney and ureter, not elsewhere classified | 1.3 |
| 62 | R32 | Unspecified urinary incontinence | 1.2 |
| 63 | G31 | Other degenerative diseases of nervous system, not elsewhere classified | 1.2 |
| 64 | Y95 | Nosocomial condition | 1.2 |
| 65 | S09 | Other and unspecified injuries of head | 1.2 |
| 66 | R45 | Symptoms and signs involving emotional state | 1.2 |
| 67 | G45 | Transient cerebral ischaemic attacks and related syndromes | 1.2 |
| 68 | Z74 | Problems related to care-provider dependency | 1.1 |
| 69 | M79 | Other soft tissue disorders, not elsewhere classified | 1.1 |
| 70 | W06 | Fall involving bed | 1.1 |
| 71 | S01 | Open wound of head | 1.1 |
| 72 | A04 | Other bacterial intestinal infections | 1.1 |
| 73 | A09 | Diarrhoea and gastroenteritis of presumed infectious origin | 1.1 |
| 74 | J18 | Pneumonia, organism unspecified | 1.1 |
| 75 | J69 | Pneumonitis due to solids and liquids | 1 |
| 76 | R47 | Speech disturbances, not elsewhere classified | 1 |
| 77 | E55 | Vitamin D deficiency | 1 |
| 78 | Z93 | Artificial opening status | 1 |
| 79 | R02 | Gangrene, not elsewhere classified | 1 |
| 80 | R63 | Symptoms and signs concerning food and fluid intake | 0.9 |
| 81 | H91 | Other hearing loss | 0.9 |
| 82 | W10 | Fall on and from stairs and steps | 0.9 |
| 83 | W01 | Fall on same level from slipping, tripping and stumbling | 0.9 |
| 84 | E05 | Thyrotoxicosis [hyperthyroidism] | 0.9 |

**eTable 3. Continued**

| **#** | **ICD-10 code** | **Description** | **Weight** |
| --- | --- | --- | --- |
| 85 | M41 | Scoliosis | 0.9 |
| 86 | R13 | Dysphagia | 0.8 |
| 87 | Z99 | Dependence on enabling machines and devices | 0.8 |
| 88 | U80 | Agent resistant to penicillin and related antibiotics | 0.8 |
| 89 | M80 | Osteoporosis with pathological fracture | 0.8 |
| 90 | K92 | Other diseases of digestive system | 0.8 |
| 91 | I63 | Cerebral Infarction | 0.8 |
| 92 | N20 | Calculus of kidney and ureter | 0.7 |
| 93 | F10 | Mental and behavioural disorders due to use of alcohol | 0.7 |
| 94 | Y84 | Other medical procedures as the cause of abnormal reaction | 0.7 |
| 95 | R00 | Abnormalities of heart beat | 0.7 |
| 96 | J22 | Unspecified acute lower respiratory infection | 0.7 |
| 97 | Z73 | Problems related to life-management difficulty | 0.6 |
| 98 | R79 | Other abnormal findings of blood chemistry | 0.6 |
| 99 | Z91 | Personal history of risk-factors, not elsewhere classified | 0.5 |
| 100 | S51 | Open wound of forearm | 0.5 |
| 101 | F32 | Depressive episode | 0.5 |
| 102 | M48 | Spinal stenosis (secondary code only) | 0.5 |
| 103 | E83 | Disorders of mineral metabolism | 0.4 |
| 104 | M15 | Polyarthrosis | 0.4 |
| 105 | D64 | Other anaemias | 0.4 |
| 106 | L08 | Other local infections of skin and subcutaneous tissue | 0.4 |
| 107 | R11 | Nausea and vomiting | 0.3 |
| 108 | K52 | Other noninfective gastroenteritis and colitis | 0.3 |
| 109 | R50 | Fever of unknown origin | 0.1 |

*Abbreviations: ICD-10: International Classification of Diseases, 10th revision.*

**eTable 4. Identification of chronic diseases during the 5 years before nursing home admission from ICD-10 diagnostic codes and drug dispensing data**

The following algorithm was adapted from ﻿Calderón-Larrañaga A, Vetrano DL, Onder G, et al. Assessing and Measuring Chronic Multimorbidity in the Older Population: A Proposal for Its Operationalization. *Journals Gerontol A Biol Sci Med Sci*. 2017;72(10):1417-1423. Compared with the original assessment tool, 4 conditions were excluded from the total number of chronic diseases (dementia, Parkinson's disease, epilepsy and chronic kidney disease) to avoid multicollinearity since these conditions are also accounted for in the Hospital Frailty Risk Score (see eTable 3).

| **Chronic Disease** | **Clinical diagnoses (ICD-10 codes)** | **Drug dispensing data** |
| --- | --- | --- |
| **Source(s) of data:** | Swedish National Patient Register | Swedish Prescribed Drug Register |
| Allergy | J30.1-J30.4; J45.0; K52.2; L20; L23; L50.0; Z51.6 |  |
| Anemia | D50-D53; D55-D59 (excl. D56.3; D59.0; D59.2; D59.3; D59.6); D60-D64 (excl. D60.1; D61.1; D61.2; D62; D64.2) | Drug indications in combination with ATC codes B03A, B03XA |
| Asthma | J45 | ATC codes R03DC; R03BC |
| Atrial fibrillation | I48 | Drug indications in combination with ATC codes C07A, B01A, C01AA05, C01D, C01B or C08D |
| Autoimmune diseases | I73.1; L10 (excl. L10.5); L12; L40; L41; L93-L95; M30-M36 (excl. M32.0; M34.2; M35.7-M35.9; M36.0; M36.1; M36.2; M36.3) | D05 |
| Blindness, visual loss | H54 (excl. H54.3); Z44.2; Z97.0 |  |
| Blood and blood forming organ diseases | D66-D69 (excl. D68.3; D68.4; D69.5); D71; D72.0; D73.0-D73.2; D74 (excl. D74.8); D75.0; D76.1; D76.3; D77; D80 (excl. D80.7); D81-D84; D86; D89 (excl. D89.1; D89.3) | Drug indication |
| Bradycardias and conduction diseases | I44.1-I44.3; I45.3; I45.5; Z95.0 | Drug indication in combination with ATC codes C01A, C01B or C07A |
| Cardiac valve diseases | I05-I08; I09.1; I09.8; I34-I38; I39.0-I39.4; Q22; Q23; Z95.2-Z95.4 | Drug indication |
| Cataract and other lens diseases | H25-H28; Q12; Z96.1 | Drug indication |
| Cerebrovascular disease | G45; G46; I60-I64; I67; I69 | Drug indication in combination with ATC codes B01A, C10, or N03AX |
| Chromosomal abnormalities | Q90-Q99 |  |
| Chronic infectious diseases | A15-A19; A30; A31; A50-A53 (excl. A51); A65-A67; A69.2; A81; B20-B24; B38.1; B39.1; B40.1; B57.2-B57.5; B65; B92; B94; J65; M86.3-M86.6 | J04A, excl. J04AB01, J04AB02, J04AB03 and J04AC |
| Chronic liver disease | B18; K70 (excl. K70.0; K70.1); K71.3-K71.5; K71.7; K72.1; K73; K74; K75.3-K75.8; K76.1; K76.6; K76.7; K77.8; Q44.6; Z94.4 | Drug indication or ATC codes A05AA02, A06AD11 |
| Chronic pancreas, biliary tract and gallbladder diseases | K80.0; K80.1; K80.2; K80.8; K81.1; K86 (excl. K86.2; K86.3; K86.9); Q44.0-Q44.5; Q45.0 | ATC code A09AA02 |
| Chronic ulcer of the skin | I83.0; I83.2; L89; L97; L98.4 |  |
| Colitis and related diseases | K52.0; K52.8; K55.1; K55.2; K57.2-K57.5; K57.8; K57.9; K58; K59.0; K59.2; K62 (excl. K62.0; K62.1; K62.5; K62.6); K63.4; K64 (excl. K64.5); |  |
| COPD, emphysema, chronic bronchitis | J41-J44; J47 | ATC code R03BB, or drug indication in combination with ATC codes H02AB, J01A, J01C, N02A, R03, or R05. |
| Deafness, hearing loss | H80; H90; H91.1; H91.3; H91.9; Q16; Z45.3; Z46.1; Z96.2; Z97.4 |  |
| Depression and mood diseases | F30-F34; F38; F39; F41.2 | Drug indication in combination with ATC codes N03A, N05AD, N05AH, N05AN, N05AX, N06A |
| Diabetes | E10; E11; E13; E14; E89.1 | ATC code A10 |
| Dorsopathies | M40-M43; M47-M53; Q67.5; Q76.4; Q76.1; |  |
| Dyslipidemia | E78 |  |
| Ear, nose, throat diseases | H60.4; H66.1-H66.3; H70.1; H71; H73.1; H74.1; H81.0; H83.1; H83.2; H95; J30.0; J31-J33; J34.1-J34.3; J35; J37; J38.0; J38.6; K05.1; K05.3; K07; K11.0; K11.7; Q30-Q32; Q35-Q38 |  |
| Esophagus, stomach and duodenum diseases | I85; I86.4; I98.2; I98.3; K21; K22.0; K22.2; K22.4; K22.5; K22.7; K23.0; K23.1; K25.4-K25.7; K26.4-K26.7; K27.4-K27.7; K28.4-K28.7; K29.3-K29.9; K31.1-K31.5; Q39; Q40; Z90.3 | ATC code A02BX, or drug indication in combination with ATC code A02 |
| Glaucoma | H40.1-H40.9 | ATC code S01ED or drug indication |
| Heart failure | I11.0; I13.0; I13.2; I27; I28.0; I42; I43; I50; I51.5; I51.7; I52.8; Z94.1; Z94.3 | Drug indication in combination with ATC codes C01, C03, C07A or C09 |
| Hematological neoplasms | C81-C96 |  |
| Hypertension | I10-I15 | Drug indication in combination with ATC codes C02, C03, C07, C08, or C09 |
| Inflammatory arthropathies | M02.3; M05-M14; M45; M46.0; M46.1; M46.8; M46.9 | ATC code M01CB, or drug indication in combination with ATC codes H02AB, L04, M04 |
| Inflammatory bowel disease | K50; K51 | ATC code A07E, or indication for Crohn’s disease |
| Ischemic heart disease | I20-I22; I24; I25; Z95.1; Z95.5 | ATC code C01DA or C01EB18, or drug indication in combination with ATC codes C01 to C10. |
| Migraine and facial pain syndromes | G43; G44.0-G44.3; G44.8; G50 | ATC code N02C or drug indication |
| Multiple sclerosis | G35 | Drug indication |
| Neurotic, stress-related and somatoform diseases | F40-F48 (excl. F43.0; F43.2) |  |
| Obesity | E66 |  |
| Osteoarthritis and other degenerative joint diseases | M15-M19; M36.2; M36.3 | Drug indication in combination with ATC code M01 to M05 |
| Osteoporosis | M80-M82 | ATC codes M05BA; M05BB; M05BX03; M05BX53, or drug indication in combination with ATC code M05 |
| Other cardiovascular diseases | I09 (excl. I09.1; I09.8); I28.1; I31.0; I31.1; I45.6; I49.5; I49.8; I70-I72 (excl. I70.2); I79.0; I79.1; I95.0; I95.1; I95.8; Q20; Q21; Q24-Q28; Z95.8; Z95.9 |  |
| Other digestive diseases | K66.0; K90.0-K90.2; K91.1; K93; Q41-Q43; R15; Z90.4; Z98.0 |  |
| Other eye diseases | H02.2-H02.5; H04 (excl. H04.3); H05 (excl. H05.0); H10.4; H17; H18.4-H18.9; H19.3; H19.8; H20.1; H21; H31.0-H31.2; H31.8; H31.9; H33; H35.2-H35.5; H35.7-H35.9; H36; H47-H49 (excl. H47.0; H47.1; H48.1); H51; Q10-Q15 (excl. Q12); Z94.7 |  |
| Other genitourinary diseases | B90.1; N20.0; N20.2; N20.9; N21.0; N21.8; N21.9; N22; N30.1-N30.4; N31; N32.0; N32.3; N32.8; N32.9; N33; N35; N39.3; N39.4; N48.0; N48.4; N48.9; N70.1; N71.1; N73.1; N73.4; N73.6; N76.1; N76.3; N81; N88; N89.5; N90.5; N95.2; Q54; Q62.0-Q62.4; Q62.7; Q62.8; Q63.8; Q63.9; Q64.0; Q64.1; Q64.3-Q64.9; Z90.6; Z90.7; Z96.0 | Drug indication |
| Other metabolic diseases | E20-E31 (excl. E23.1; E24.2; E24.4; E27.3; E30); E34 (excl. E34.3; E34.4); E35 (excl. E35.0); E40-E46 (excl. E44.1); E64; E70-E72; E74-E77; E79 (excl. E79.0); E80 (excl. E80.4); E83-E89 (excl. E86; E87; E88.3; E89.0; E89.1); K90.3; K90.4; K90.8; K90.9; K91.2; M83; M88; N25 |  |
| Other musculoskeletal and joint diseases | B90.2; M21.2-M21.9; M22-M24; M25.2; M25.3; M35.7; M61; M65.2-M65.4; M70.0; M72.0; M72.2; M72.4; M75.0; M75.1; M75.3; M75.4; M79.7; M84.1; M89; M91; M93; M94; M96; M99; S38.2; S48; S58; S68; S78; S88; S98; T05; T09.6; T11.6; T13.6; T14.7; T90-T98; Q65; Q66; Q68; Q71-Q74; Q77; Q78; Q79.6; Q79.8; Q87; Z44.0; Z44.1; Z89.1-Z89.9; Z94.6; Z96.6; Z97.1 | Drug indication |
| Other neurological diseases | B90.0; D48.2; G04.1; G09-G14 (excl. G13.0; G13.1); G24-G26 (excl. G25.1; G25.4; G25.6); G32; G37; G51-G53 (excl. G51.0); G70; G71; G72.3-72.9; G73 (excl. G73.2-G73.4); G80-G83 (excl. G83.8); G90; G91; G93.8; G93.9; G95; G99; M47.1; Q00-Q07; Q76.0 |  |
| Other psychiatric and behavioral diseases | F04; F06; F07; F09; F10.2; F10.6; F10.7; F11.2; F11.6; F11.7; F12.2; F12.6; F12.7; F13.2; F13.6; F13.7; F14.2; F14.6; F14.7; F15.2; F15.6; F15.7; F16.2; F16.6; F16.7; F17.2; F17.6; F17.7; F18.2; F18.6; F18.7; F19.2; F19.6; F19.7; F50; F52; F60-F63; F68; F70-F89; F95; F99 | ATC code N07BB, or indication for alcohol-related dependence |
| Other respiratory diseases | B90.9; E66.2; J60-J67; J68.4; J70.1; J70.3; J70.4; J84; J92; J94.1; J95.3; J95.5; J96.1; J98 (excl. J98.1); Q33; Q34; Z90.2; Z94.2; Z94.3; Z96.3 |  |
| Other skin diseases | L13; L28; L30.1; L43 (excl. L43.2); L50.8; L58.1; L85; Q80; Q81; Q82.1; Q82.2; Q82.9 |  |
| Peripheral neuropathy | B91; G54-G60; G62.8; G62.9; G63 (excl. G63.1); M47.2; M53.1; M54.1 | Drug indication |
| Peripheral vascular disease | I70.2; I73 (excl. I73.1; I73.8); I79.2; I79.8 | B01AC23 |
| Prostate diseases | N40; N41.1; N41.8 | ATC code G04C (excl. G04CB), or drug indication |
| Schizophrenia and delusional diseases | F20; F22; F24; F25; F28 | Drug indication in combination with ATC code N05 |
| Sleep disorders | G47; F51.0-F51.3 |  |
| Solid neoplasms | All C (excl. C81-C96); D00-D09; D32.0; D32.1; D32.9; D33.0-D33.4; Q85 | ATC code L01X, or drug indication |
| Thyroid disease | E00-E03 (excl. E03.5); E05; E06.2; E06.3; E06.5; E07; E35.0; E89.0 | ATC codes H03AA; H03B; or drug indication in combination with ATC code H03 |
| Venous and lymphatic diseases | I78.0; I83; I87; I89; I97.2; Q82.0 |  |

**Appendix. Sensitivity analyses**

We conducted a series of *pre-specified* sensitivity analyses to address the main sources of bias that could arise in our cohort. First, we restricted the analysis to nursing home residents admitted in or after June 2013 to reduce the possibility of exposure misclassification (i.e. using a 6-month washout period to remove older persons who had in fact already been admitted to a nursing home in the past). Second, we restricted our analyses to nursing home residents who had complete and uninterrupted follow-up data in the Social Services Register (i.e. less than 25% of months at-risk with missing information). Third, we evaluated whether our main analyses were affected by exclusion of nursing home residents who died within 3 months of their admission by re-including them. Conversely, we tested the robustness of our findings by restricting the analysis to newly admitted residents who survived at least 6 months in order to assess whether the observed association between education and unplanned hospitalisations was driven by residents admitted with serious illness and poor prognosis. In another set of sensitivity analyses, we moved the date of cohort entry (‘time zero’) from the first day of the month immediately *after* nursing home admission to the midmonth date of the month in question (e.g. 16 March instead of 1 April for a person admitted in March). This was done in an effort to address some of the inaccuracy around the exact date of nursing home admission. We also calculated the E-value to assess the robustness of our results to unmeasured confounding, by using the methodology of VanderWeele and Ding (*Ann Intern Med*. 2017;167(4):268). Finally, we examined the association between socioeconomic position and the incidence of planned hospitalisations to serve as a positive control outcome, based on the assumption that older adults with higher education may have preferences and values that makes them more prone to choose elective surgeries and procedures.

We also conducted two sets of *post-hoc* analyses, which were not specified *a priori* but instead decided during the course of the study. First, we examined whether operationalizing socioeconomic position with the relative level of disposable income instead of the highest educational attainment had any influence on our findings. For this purpose, we categorized newly admitted nursing home residents across sex and age-specific (1-year bands) quartiles of disposable income based on data from the longitudinal integration database for health insurance and labour market studies (LISA). Second, we assessed the presence of multiplicative interaction effects between education and levels of income.

**eTable 5. Mortality rates by level of education**

|  | **Primary education** | **Secondary education** | **Tertiary education** |
| --- | --- | --- | --- |
| No. of nursing home residents | 20 614 | 15 339 | 4 592 |
| Person-years of follow-up | 30 465 | 22 658 | 6 724 |
| No. of deaths | 8 532 | 6 002 | 1 797 |
| Mortality rate per 100 person-years (95% CI) | 28.0 (27.4-28.6) | 26.5 (25.8-27.2) | 26.7 (25.5-28.0) |
| Adjusted incidence rate ratio (95% CI)^a^ | 1 | 0.96 (0.93-0.99) | 0.93 (0.88-0.99) |

^a^ *Poisson regression model adjusted for age, sex, marital status, frailty, number of chronic diseases, number of drugs, and number of days of inpatient stay before nursing home admission.*

**eTable 6. Sensitivity analysis: level of education and risk of unplanned hospitalisation among newly admitted nursing home residents**

|  | **Primary education** | **Secondary education** | **Tertiary education** |
| --- | --- | --- | --- |
| No. of nursing home residents | 20 614 | 15 339 | 4 592 |
| Person-years of follow-up | 30 169 | 22 429 | 6 660 |
| No. of unplanned hospitalisations | 16 621 | 12 425 | 3 592 |
| Rate per 100 person-years (95% CI) | 55.1 (54.3-55.9) | 55.4 (54.4-56.4) | 53.9 (52.2-55.7) |
| Adjusted incidence rate ratio (95% CI) |  |  |  |
| Zero-inflated Poisson regression (main model)^a^ | 1 | 0.99 (0.96-1.02) | 0.96 (0.92-1.00) |
| E-value^b^ |  | 1.11 | 1.25 |
| Poisson regression^c^ | 1 | 0.99 (0.96-1.01) | 0.95 (0.91-0.98) |
| E-value^b^ |  | 1.11 | 1.29 |
| Zero-inflated negative binomial regression^d^ | 1 | 0.98 (0.95-1.02) | 0.94 (0.89-0.99) |
| E-value^b^ |  | 1.16 | 1.32 |

*Abbreviations: CI, Confidence Interval*

*^a^ Zero-inflated Poisson regression model adjusted for age, sex, marital status, frailty, number of chronic diseases, number of drugs, and number of days of inpatient stay before nursing home admission. The ‘zero’ model was adjusted for the number of chronic diseases and the number of days of inpatient stay before nursing home admission. Please note that this model is identical to the one reported in Table 2.*

*^b^ E-values are reported for point estimates. Interpretation: we found that the observed adjusted incidence rate ratio (IRR) for unplanned hospitalisation of 0.96 (tertiary vs. primary education) could be fully explained away by an unmeasured confounder that was associated with both education and risk of unplanned hospitalisation by an IRR of 1.25 either above and beyond the measured confounders, but a weaker confounder could not do so.*

*^c^ Poisson regression model adjusted for age, sex, marital status, frailty, number of chronic diseases, number of drugs, and number of days of inpatient stay before nursing home admission.*

*^d^ Zero-inflated negative binomial regression model adjusted for age, sex, marital status, frailty, number of chronic diseases, number of drugs, and number of days of inpatient stay before nursing home admission. The ‘zero’ model was adjusted for the number of chronic diseases.*

**eTable 7. Sensitivity analysis: level of education and cumulative length of stay of unplanned hospitalisations**

|  | **Primary education** | **Secondary education** | **Tertiary education** |
| --- | --- | --- | --- |
| No. of nursing home residents | 20 614 | 15 339 | 4 592 |
| Person-years of follow-up | 30 465 | 22 658 | 6 724 |
| No. of days spent in hospital | 101 846 | 78 406 | 21 941 |
| Fraction of time spend in hospital (%) | 0.9% | 0.9% | 0.9% |
| Adjusted incidence rate ratio (95% CI) |  |  |  |
| Zero-inflated Poisson regression (main model)^a^ | 1 | 1.03 (1.02-1.04) | 1.01 (0.99-1.02) |
| E-value^b^ |  | 1.21 | 1.11 |
| Poisson regression^c^ | 1 | 1.01 (0.98-1.04) | 0.94 (0.90-0.99) |
| E-value^b^ |  | 1.11 | 1.32 |

*Abbreviations: CI, Confidence Interval*

*^a^ Zero-inflated Poisson regression model adjusted for age, sex, marital status, frailty, number of chronic diseases, number of drugs, and number of days of inpatient stay before nursing home admission. The ‘zero’ model was adjusted for the number of chronic diseases and the number of days of inpatient stay before nursing home admission. Please note that this model is identical to the one reported in Table 2.*

*^b^ E-values are reported for point estimates. Interpretation: we found that the observed adjusted incidence rate ratio (IRR) for cumulative length of stay of unplanned hospitalisation of 1.01 (tertiary vs. primary education) could be fully explained away by an unmeasured confounder that was associated with both education and risk of unplanned hospitalisation by an IRR of 1.11 either above and beyond the measured confounders, but a weaker confounder could not do so.*

*^c^ Poisson regression model adjusted for age, sex, marital status, frailty, number of chronic diseases, number of drugs, and number of days of inpatient stay before nursing home admission.*

**eTable 8. Sensitivity analysis: level of education and risk of *planned* hospitalisation among newly admitted nursing home residents**

|  | **Primary education** | **Secondary education** | **Tertiary**  **education** |
| --- | --- | --- | --- |
| No. of nursing home residents | 20 614 | 15 339 | 4 592 |
| Person-years of follow-up | 30 169 | 22 429 | 6 660 |
| No. of planned hospitalisations | 1 062 | 1 023 | 275 |
| Rate per 100 person-years (95% CI) | 3.5 (3.3-3.7) | 4.6 (4.3-4.8) | 4.1 (3.7-4.6) |
| Adjusted incidence rate ratio (95% CI) |  |  |  |
| Zero-inflated Poisson regression^a^ | 1 | 1.23 (1.11-1.36) | 1.14 (0.98-1.33) |
| Poisson regression^b^ | 1 | 1.21 (1.11-1.32) | 1.09 (0.95-1.24) |

*Abbreviations: CI, Confidence Interval*

*^a^ Zero-inflated Poisson regression model adjusted for age, sex, marital status, frailty, number of chronic diseases, number of drugs, and number of days of inpatient stay before nursing home admission. The ‘zero’ model was adjusted for the number of chronic diseases and the number of days of inpatient stay before nursing home admission.*

*^b^ Poisson regression model adjusted for age, sex, marital status, frailty, number of chronic diseases, number of drugs, and number of days of inpatient stay before nursing home admission.*

**eTable 9. Sensitivity analysis: income quartile and risk of unplanned hospitalisation among newly admitted nursing home residents (Poisson regression)**

|  | **Lowest income quartile** | **2^nd^ income quartile** | **3^rd^ income quartile** | **Highest income quartile** |
| --- | --- | --- | --- | --- |
| No. of nursing home residents | 9 324 | 10 375 | 11 003 | 9 821 |
| Person-years of follow-up | 13 936 | 15 291 | 15 886 | 14 115 |
| No. of unplanned hospitalisations | 7 465 | 8 403 | 9 145 | 7 603 |
| Rate per 100 person-years (95% CI) | 53.6 (52.4-54.8) | 55.0 (53.8-56.1) | 57.6 (56.4-58.8) | 53.9 (52.7-55.1) |
| Adjusted incidence rate ratio (95% CI)^a^ | 1 | 0.98 (0.95-1.01) | 1.01 (0.98-1.04) | 0.96 (0.93-0.99) |

*Abbreviations: CI, Confidence Interval*

*A total of 22 individuals had no data regarding their annual income and were thus excluded from the analysis. Income quartiles were calculated for each sex-age stratum separately.*

*^a^ Poisson regression model adjusted for age, sex, marital status, frailty, number of chronic diseases, number of drugs, and number of days of inpatient stay before nursing home admission.*

**eTable 10. Sensitivity analysis: level of education and risk of unplanned hospitalisations among nursing home residents admitted in or after June 2013 (6-month washout)**

|  | **Primary education** | **Secondary education** | **Tertiary**  **education** |
| --- | --- | --- | --- |
| No. of nursing home residents | 18 535 | 13 914 | 4 172 |
| Person-years of follow-up | 26 165 | 19 638 | 5 854 |
| No. of unplanned hospitalisations | 14 481 | 10 950 | 3 152 |
| Rate per 100 person-years (95% CI) | 55.3 (54.4-56.3) | 55.8 (54.7-56.8) | 53.8 (52.0-55.8) |
| Adjusted incidence rate ratio (95% CI)^a^ | 1 | 0.99 (0.96-1.02) | 0.96 (0.92-1.00) |

*Abbreviations: CI, Confidence Interval*

*^a^ Zero-inflated Poisson regression model adjusted for age, sex, marital status, frailty, number of chronic diseases, number of drugs, and number of days of inpatient stay before nursing home admission. The ‘zero’ model was adjusted for the number of chronic diseases and the number of days of inpatient stay before nursing home admission.*

**eTable 11. Sensitivity analysis: level of education and risk of unplanned hospitalisations among nursing home residents with complete and uninterrupted follow-up data in the Social Services Register**

|  | **Primary education** | **Secondary education** | **Tertiary**  **education** |
| --- | --- | --- | --- |
| No. of nursing home residents | 18 785 | 14 095 | 4 225 |
| Person-years of follow-up | 27 593 | 20 642 | 6 148 |
| No. of unplanned hospitalisations | 14 929 | 11 069 | 3 269 |
| Rate per 100 person-years (95% CI) | 54.1 (53.2-55.0) | 53.6 (52.6-54.6) | 53.2 (51.4-55.0) |
| Adjusted incidence rate ratio (95% CI)^a^ | 1 | 0.98 (0.95-1.01) | 0.97 (0.93-1.01) |

*Abbreviations: CI, Confidence Interval*

*^a^ Zero-inflated Poisson regression model adjusted for age, sex, marital status, frailty, number of chronic diseases, number of drugs, and number of days of inpatient stay before nursing home admission. The ‘zero’ model was adjusted for the number of chronic diseases and the number of days of inpatient stay before nursing home admission.*

**eTable 12. Sensitivity analysis: level of education and risk of unplanned hospitalisation during follow-up among all newly admitted residents (including those who died within 3 months)**

|  | **Primary education** | **Secondary education** | **Tertiary**  **education** |
| --- | --- | --- | --- |
| No. of nursing home residents | 22 274 | 16 557 | 4 955 |
| Person-years of follow-up | 30 414 | 22 608 | 6 715 |
| No. of unplanned hospitalisations | 17 566 | 13 175 | 3 792 |
| Rate per 100 person-years (95% CI) | 57.8 (56.9-58.6) | 58.3 (57.3-59.3) | 56.5 (54.7-58.3) |
| Adjusted incidence rate ratio (95% CI)^a^ | 1 | 0.99 (0.97-1.02) | 0.96 (0.92-1.00) |

*Abbreviations: CI, Confidence Interval*

*^a^ Zero-inflated Poisson regression model adjusted for age, sex, marital status, frailty, number of chronic diseases, number of drugs, and number of days of inpatient stay before nursing home admission. The ‘zero’ model was adjusted for the number of chronic diseases and the number of days of inpatient stay before nursing home admission.*

**eTable 13. Sensitivity analysis: level of education and risk of unplanned hospitalisation during follow-up among nursing home residents who survived at least 6 months (Poisson regression)**

|  | **Primary education** | **Secondary education** | **Tertiary  education** |
| --- | --- | --- | --- |
| No. of nursing home residents | 18 883 | 14 032 | 4 232 |
| Person-years of follow-up | 29 566 | 21 980 | 6 535 |
| No. of unplanned hospitalisations | 15 318 | 11 376 | 3 324 |
| Rate per 100 person-years (95% CI) | 51.8 (51.0-52.6) | 51.8 (50.8-52.7) | 50.9 (49.2-52.6) |
| Adjusted incidence rate ratio (95% CI)^a^ | 1 | 0.98 (0.95-1.01) | 0.97 (0.93-1.01) |

*Abbreviations: CI, Confidence Interval*

*^a^ Zero-inflated Poisson regression model adjusted for age, sex, marital status, frailty, number of chronic diseases, number of drugs, and number of days of inpatient stay before nursing home admission. The ‘zero’ model was adjusted for the number of chronic diseases and the number of days of inpatient stay before nursing home admission.*

**eTable 14. Sensitivity analysis: level of education and risk of unplanned hospitalisation during follow-up, with mid-month approximation of the date of admission**

|  | **Primary education** | **Secondary education** | **Tertiary**  **education** |
| --- | --- | --- | --- |
| No. of nursing home residents | 20 614 | 15 339 | 4 592 |
| Person-years of follow-up | 31 008 | 23 040 | 6 840 |
| No. of unplanned hospitalisations | 18 224 | 13 777 | 3 997 |
| Rate per 100 person-years (95% CI) | 58.8 (57.9-59.6) | 59.8 (58.8-60.8) | 58.4 (56.6-60.3) |
| Adjusted incidence rate ratio (95% CI)^a^ | 1 | 1.00 (0.97-1.02) | 0.97 (0.93-1.01) |

*Abbreviations: CI, Confidence Interval*

*^a^ Zero-inflated Poisson regression model adjusted for age, sex, marital status, frailty, number of chronic diseases, number of drugs, and number of days of inpatient stay before nursing home admission. The ‘zero’ model was adjusted for the number of chronic diseases and the number of days of inpatient stay before nursing home admission.*

**eTable 15. Multiplicative interaction between education and income**

|  | IRR (95% CI) for the main effect estimate | IRR (95% CI) for the interaction effect |
| --- | --- | --- |
| **Lowest income quartile** |  |  |
| Primary education | 1 | – |
| Secondary education | 0.96 (0.91–1.01) | – |
| Tertiary education | 0.94 (0.82–1.06) | – |
| **2^nd^ income quartile** |  |  |
| Primary education | 1 | 1 |
| Secondary education | 0.99 (0.93–1.04) | 1.02 (0.95–1.10) |
| Tertiary education | 0.93 (0.83–1.05) | 0.99 (0.83–1.17) |
| **3^rd^ income quartile** |  |  |
| Primary education | 1 | 1 |
| Secondary education | 1.01 (0.96–1.06) | 1.04 (0.97–1.12) |
| Tertiary education | 0.99 (0.91–1.07) | 1.05 (0.90–1.21) |
| **Highest income quartile** |  |  |
| Primary education | 1 | 1 |
| Secondary education | 1.04 (0.98–1.11) | 1.08 (1.00–1.17) |
| Tertiary education | 1.02 (0.95–1.09) | 1.09 (0.94–1.26) |

*Abbreviations: CI, Confidence Interval*

*Zero-inflated Poisson regression models are adjusted for age, sex, marital status, frailty, number of chronic diseases, number of drugs, and number of days of inpatient stay before nursing home admission. The ‘zero’ model was adjusted for the number of chronic diseases and the number of days of inpatient stay before nursing home admission.*

**eTable 16. Subgroup analysis: level of education and risk of unplanned hospitalisation among newly admitted nursing home residents, stratified by sex**

|  | **Men** | | |  | **Women** | | |
| --- | --- | --- | --- | --- | --- | --- | --- |
|  | **Primary education** | **Secondary education** | **Tertiary education** |  | **Primary education** | **Secondary education** | **Tertiary education** |
| **No. of nursing home residents** | 6 652 | 5 116 | 1 970 |  | 13 962 | 10 223 | 2 622 |
| **Unplanned hospitalisations** |  |  |  |  |  |  |  |
| Person-years of follow-up | 9 139 | 7 012 | 2 688 |  | 10 604 | 7 725 | 1 842 |
| No. of unplanned hospitalisations | 6 017 | 4 700 | 1 750 |  | 21 030 | 15 418 | 3 972 |
| Rate per 100 person-years (95% CI) | 65.8 (64.2-67.5) | 67.0 (65.1-69.0) | 65.1 (62.1-68.2) |  | 50.4 (49.5-51.4) | 50.1 (49.0-51.2) | 46.4 (44.3-48.5) |
| Unadjusted incidence rate ratio (95% CI) | 1 | 1.05 (1.00–1.09) | 1.03 (0.97–1.10) |  | 1 | 0.99 (0.95–1.02) | 0.93 (0.88–0.98) |
| Adjusted incidence rate ratio (95% CI)^a^ | 1 | 1.01 (0.97-1.06) | 1.01 (0.95-1.07) |  | 1 | 0.98 (0.94-1.01) | 0.93 (0.88-0.98) |
| E-value^b^ | - | 1.11 | 1.11 |  | - | 1.16 | 1.36 |
| **Number of days of unplanned admissions** |  |  |  |  |  |  |  |
| Person-years of follow-up | 9 246 | 7 098 | 2 720 |  | 21 219 | 15 560 | 4 004 |
| No. of days hospitalized | 36 720 | 29 619 | 10 975 |  | 65 126 | 48 787 | 10 966 |
| Fraction of time spend in hospital (%) | 1.1% | 1.1% | 1.1% |  | 0.8% | 0.9% | 0.7% |
| No. days hospitalized per person-year (95% CI) | 4.0 (3.9-4.0) | 4.2 (4.1-4.2) | 4.0 (4.0-4.1) |  | 3.1 (3.0-3.1) | 3.1 (3.1-3.2) | 2.7 (2.7-2.8) |
| Unadjusted incidence rate ratio (95% CI) | 1 | 1.08 (1.07–1.10) | 1.08 (1.06–1.11) |  | 1 | 1.03 (1.02–1.05) | 0.97 (0.95–0.99) |
| Adjusted incidence rate ratio (95% CI)^a^ | 1 | 1.06 (1.04-1.07) | 1.06 (1.04-1.09) |  | 1 | 1.02 (1.01-1.03) | 0.96 (0.94-0.98) |
| E-value^b^ | - | 1.31 | 1.31 |  | - | 1.16 | 1.25 |

*Abbreviations: CI, Confidence Interval*

*^a^ Zero-inflated Poisson regression model adjusted for age, sex, marital status, frailty, number of chronic diseases, number of drugs, and number of days of inpatient stay before nursing home admission. The ‘zero’ model was adjusted for the number of chronic diseases and the number of days of inpatient stay before nursing home admission.*

*^b^ E-values are reported for point estimates. Interpretation: we found that the observed adjusted incidence rate ratio (IRR) for unplanned hospitalisation of 1.01 among male residents (tertiary vs. primary education) could be fully explained away by an unmeasured confounder that was associated with both education and risk of unplanned hospitalisation by an IRR of 1.11 either above and beyond the measured confounders, but a weaker confounder could not do so.*
